# Belladonna as Prophylactic in Scarlatina

**Published:** 1844-04

**Authors:** 


### PRACTICAL MEDICINE.

### BELLADONNA AS PROPHYLACTIC IN SCARLATINA.

An epidemic Scarlatina has been for some time prevailing, in Chicago. The mortality has been great, but not so much so as is generally supposed by the citizens. The number of deaths, from the disease and sequelæ, in the months of January and February, during which time the disease was most malignant, was 27. We have heard of but three fatal cases from that time to the present. The number of cases occurring we have been unable to ascertain, as no general record was kept by the physicians of the place.

During the prevalence of the epidemic, the prophylactic powers of the Belladonna have been tested in a number of cases, and without success. We have been informed of fourteen instances in which the supposed preventive was taken, and in which the patients afterwards took the disease. Of these, six cases were fatal. To what extent it was given, or how long before the accession of the disease we have no means of ascertaining.

As public attention has been called to this subject, the following opinions of eminent authorities may be interesting.

Dr. Pereira remarks,—" Bearing in mind the well known capriciousness evinced by scarlet fever, (as indeed by other contagious disorders) in regard to the subjects of its attacks, and the large number of those who, though exposed to its influence, escape, the best evidence hitherto adduced in favor of the notion must be admitted to be inconclusive. While, therefore, the facts brought forward in favour of the existence of this prophylactic power are only negative, those which can be adduced against it are positive. For I conceive twenty cases of failure are more conclusive against the opinion here referred to, than one thousand of non-occurrence are in favor of it. Now Lehman, Barth, Murbeck, Hoffman, and many others that I could refer to, declare that it has failed in their hands to evince its prophylactic powers. In this country (England) we have had no extended series of observations to quote; but the cases which I am acquainted with are decidedly against the efficacy of the remedy. A remarkable failure is mentioned by Dr. Sigmond, of a family of eleven persons who took the supposed specific, yet every individual contracted the disease." [Pereira's Mat. Med. and Ther., vol. 2, p. 307.]

In a review of " A practical treatise of the diseases of children, by D. Francis Condie, M. D. &c.," in the last number (Jan. '44) of the American Journal, we find the following: " Of the prophylactic powers of Belladonna in Scarlatina, Dr. Condie gives the following opinion."

"We have in repeated instances, tested the prophylactic powers of Belladonna, but although redness and dryness of the throat, and a diffuse scarlet efflorescence were produced in a majority of cases, we never found it, in any, to produce the slightest effect in mitigating the character or preventing the occurrence of Scarlatina. The experiments were made during the prevalence of the disease, and in numerous instances the subjects of them were attacked. In one case the efflorescence was kept up by the use of the Belladonna, for forty-eight hours; in a week afterwards this individual took the disease, in its most violent form, and died on the fourth day."

